# Spatiotemporally explicit model averaging for forecasting of Alaskan groundfish catch

**DOI:** 10.1002/ece3.4488

**Published:** 2018-12-07

**Authors:** Hannah E. Correia

**Affiliations:** ^1^ Department of Biological Sciences Auburn University Auburn Alabama; ^2^ Norwegian Institute for Nature Research (NINA) Tromsø Norway

**Keywords:** forecast, model averaging, multimodel inference, spatio‐temporal

## Abstract

Fisheries management is dominated by the need to forecast catch and abundance of commercially and ecologically important species. The influence of spatial information and environmental factors on forecasting error is not often considered. I propose a forecasting method called spatiotemporally explicit model averaging (STEMA) to combine spatial and temporal information through model averaging. I examine the performance of STEMA against two popular forecasting models and a modern spatial prediction model: the autoregressive integrated moving averages with explanatory variables (ARIMAX) model, the Bayesian hierarchical model, and the varying coefficient model. I focus on applying the methods to four species of Alaskan groundfish for which catch data are available. My method reduces forecasting errors significantly for most of the tested models when compared to ARIMAX, Bayesian, and varying coefficient methods. I also consider the effect of sea surface temperature (SST) on the forecasting of catch, as multiple studies reveal a potential influence of water temperature on the survival and growth of juvenile groundfish. For most of the preferred models, inclusion of SST in the model improved forecasting of catch. It is advisable to consider both spatial information and relevant environmental factors in forecasting models to obtain more accurate projections of population abundance. The STEMA method is capable of accounting for spatial information in forecasting and can be applied to various types of data because of its flexible varying coefficient model structure. It is therefore a suitable forecasting method for application to many fields including ecology, epidemiology, and climatology.

## 1 INTRODUCTION

Forecasting is a vital component of fisheries management and furnishes necessary input for management decisions. However, forecasting models are often based on simplistic time series trend analyses, which do not capture spatial information. Parametric time series methods such as autoregressive integrated moving averages (ARIMA) models provide accurate forecasts when the trend is consistent, or many time points are supplied (Box & Jenkins, 1970). These time series methods often fail when there are insufficient measures over time or the response fluctuates with little apparent trend (Koutroumanidis, Iliadis, & Sylaios, 2006; Stergiou & Christou, 1996). Another issue with these models is the inclusion of covariates. Often the covariates of interest must be forecast individually and the relationship between those predictors and the response are assumed to be linear (Box, Jenkins, & Reinsel, 2013). This is an unrealistic assumption for recent climate data that exhibit anthropogenic‐driven trends where the pattern of the relationship between predictors and response can change over time. Additionally, time series methods are unable to consider covariates that also vary over space.

Varying coefficient models offer a flexible modeling structure which allows for nonlinear relationships between predictors and the response; these models are also capable of handling covariates that change over space and time (Augustin, Trenkel, Wood, & Lorance, 2013; Hastie & Tibshirani, 1993; Phillips, Ciannelli, Brodeur, Pearcy, & Childers, 2014). Unlike time series models, prediction in varying coefficient models is limited to the model structure and cannot produce forecasts beyond the time range of the data. It would thus be ideal to combine the nonlinear spatial information from varying coefficient models with the forecasting capabilities of time series methods. Model averaging allows the combination of information from multiple models to inform the predictions of a response while accounting for model uncertainty. The models are often weighted according to best fit using the Akaike information criterion (AIC) or the Bayesian information criterion (BIC) (Buckland, Burnham, & Augustin, 1997) in the aggregation process; however, other criteria for weighting are also possible (Hansen, 2007; Raftery, Gneiting, Balabdaoui, & Polakowski, 2005). I propose a methodology based on a combination of information gained through the flexibility of varying coefficient models with the trend analyses of ARIMA models to obtain predictions for catch rates at specific locations, thereby creating predicted species distributions. The method takes advantage of varying coefficient models and model averaging while refining these for use in spatio‐temporal forecasting. My technique is the result of complex leave‐one‐out construction procedures used to create forecast stability. This procedure provides prediction errors that are used as weights for model averaging.

Water temperature has effects on the growth, survival, and behavior of juvenile fish. Survival of juveniles to reproductive age is a key indicator of population maintenance and growth. Fisheries management and restoration strategies are keen to monitor recruitment and abundance for target species. However, information on reproductive success and recruitment to model population abundance are often lacking for deep‐water marine species. Catch rates are related to population size and are commonly used in fisheries management as an index of abundance (Battaile & Quinn, 2004; Council, 2000; Ricker, 1975). Many deep‐water species move inshore to reproduce, and thus, offspring are affected by the temperature of surface waters in the first few years of life when they are sensitive to environmental extremes. Changes in SST affect plankton availability, distribution, and composition, which are an important nutrition resource for deep‐water species and act as a carbon sink (Brierley & Kingsford, 2009). Additionally, SST acts as a proxy for many other oceanic processes, affecting currents, ocean mixing, and sea ice retreat, all of which have effects on both fish biomass (Bouchard & Fortier, 2008; Hunt et al., 2002) and catch (Cheung et al., 2009; Kim et al., 2012; Monllor‐Hurtado, Pennino, & Sanchez‐Lizaso, 2017). Further, it is evident that management strategies must now consider temperature trends in order for managers to provide accurate long‐term advice (Biswas, Svirezhev, & Bala, 2005; Ianelli, Hollowed, Haynie, Mueter, & Bond, 2011; Vaidyanathan, 2017). Still, current management implementation rarely includes ecosystem processes that have been shown to affect fish stock productivity (Skern‐Mauritzen et al., 2015). It is therefore meaningful to consider SST in predicting catch, particularly in studies where recruitment information is not available or difficult to assess. While adding additional variables will improve model fit, it will not necessarily improve prediction. Thus, I assess whether prediction of catch is improved through the inclusion of SST.

## 2 BACKGROUND

Prediction of fish abundance and catch is crucial in creating management strategies for commercially important species, particularly in oceans with high system variability. The northern Pacific Ocean has undergone several recent regime shifts that affect marine groups differently (Napp & Hunt, 2001). Changes in abundance and population dynamics of various marine fishes, including salmon, cod, halibut, and sardines, have manifested in response to climatic regime shifts in the past century (Benson & Trites, 2002; Möllmann & Diekmann, 2012; Noakes & Beamish, 2009). Abrupt changes in climatic cycles via persistent, area‐specific shifts in trends of water temperature, ocean currents, and primary production create profound changes in the marine ecosystem, though the precise mechanisms through which these changes occur are still not well understood (Anderson & Piatt, 1999; Francis, Hare, Hollowed, & Wooster, 1998). Sea surface temperature (SST) is a simple measure to obtain. It acts as an easily identifiable representative for more complex relationships between oceanic and atmospheric conditions that precede or accompany marine regime shifts (Möllmann, Folke, Edwards, & Conversi, 2015; deYoung et al., 2008). Consequently, SST is the most commonly used environmental variable considered when modeling fish catch and abundance in the wild. However, not many studies consider the effect of SST on the forecasting of fish catch, especially in management settings. Water temperature has also been shown to affect the feeding motivation, metabolism, reproduction, and behavior of many fish species (Donelson, Munday, McCormick, Pankhurst, & Pankhurst, 2010; Pörtner et al., 2001), which in turn influences recruitment and abundance. Increased water temperatures due to climate change are therefore likely to affect the amount and composition of aquatic species in northern latitudes (Pörtner & Knust, 2007; Sharma, Jackson, Minns, & Shuter, 2007). Along with increased water temperatures, climate variability is expected to increase as a result of climate change (Easterling et al., 2000; Timmermann et al., 1999). Anomalous oceanic conditions brought about by persistent changes in atmospheric patterns, such as the warm SST anomaly known as “the Blob” in the Northern Pacific Ocean (Tseng, Ding, & Menghuang, 2017), have effects on regional weather and impacts on coastal and deep‐water fisheries operations as well as the composition of ecosystems (Bond, Cronin, Freeland, & Mantua, 2015). Extreme changes in the marine environment that often accompany ocean anomalies are more detrimental to juvenile fish and can affect their recruitment into the adult population (Baumann et al., 2006; Beaugrand, Brander, Alistair Lindley, Souissi, & Reid, 2003; Stige, Ottersen, Brander, Chan, & Stenseth, 2006).

Three commercially important groundfish species, Pacific cod (*Gadus macrocephalus*), Pacific halibut (*Hippoglossus stenolepis*), and sablefish (*Anoplopoma fimbria*), and the most abundant groundfish species, giant grenadier (*Albatrossia pectoralis*), are located within the north Pacific ecosystem. The commercial fisheries of Pacific cod, Pacific halibut, and sablefish are predominantly or solely longline, and catch of giant grenadier is predominantly through bycatch on sablefish longlines (Goen & Erikson, 2017; NPFMC, 2017; Rodgveller, Lunsford, & Fujioka, 2008). Pacific halibut, Pacific cod, and sablefish also have management guidelines in effect that would likely benefit from new and more accurate prediction techniques. Winter ocean conditions in the northeast Pacific Ocean have been linked to recruitment in groundfish stocks (Hollowed & Wooster, 1992; Schirripa & Colbert, 2006). Studies on juveniles of these four species show that increased water temperatures affect behavioral responses, growth, and survival (Laurel, Spencer, Iseri, & Copeman, 2016; Sogard & Olla, 2001; Stoner, Ottmar, & Hurst, 2006; Stoner & Sturm, 2004). No laboratory studies have been conducted on the temperature tolerances of giant grenadier. Sablefish and giant grenadier are known to compete for baited hooks in longline surveys (Rodgveller et al., 2008). These results indicate that giant grenadier may inhabit similar temperature zones as sablefish. This highlights the need to understand the relationship of temperature to an apex deep‐water predator likely to be the most abundant fish in the northern Pacific (Rodgveller & Hulson, 2014).

Climate change is characterized in many areas of the globe as a consistent warming trend which favors acclimation in fishes (Crozier & Hutchings, 2014). Variability in global climate systems is also increasing the occurrence of extreme climate events and changing marine ecosystems dramatically and suddenly (Hoegh‐Guldberg & Bruno, 2010; Walther et al., 2002). If oceanic conditions continue to experience increased variability and instability, persistent changes to the physiology of fishes as a result of acclimatization are likely to translate into reduced phenotypic plasticity (Reed, Schindler, & Waples, 2011; Seebacher, White, & Franklin, 2014). Pacific cod displayed “cold‐adapted” responses in hatching, growth rates, and mortality when sampled from the coldest cohort in three decades (Hurst, Munch, & Lavelle, 2012). This illustrates that groundfish from a cohort experiencing more extreme temperature changes, either anomalously cold or warm, may be at a disadvantage when experiencing the opposing extreme conditions to which they experienced when hatching. The effect is likely to be pronounced if an intensely cold year during the hatching of a cohort is followed by an extremely warm year (or vice versa) when those fish are still in their vulnerable juvenile state. It is therefore important to gain a greater understanding of the effects of temperature on commercially and ecologically important species such as those discussed here.

## 3 DATA

The data for this study were collated from two datasets provided by the National Oceanic and Atmospheric Administration (NOAA). Of primary use were the annual longline survey data of the Marine Ecology and Stock Assessment (MESA) Program conducted by the Auke Bay Laboratories in Alaska (Alaska Fisheries Science Center, NOAA, 2015). The MESA Program has performed longline surveys independently since 1979, dropping baited lines at specific locations (“stations’’) off the coast of Alaska to collect information on groundfish species. Seven major groundfish species are surveyed in the MESA Program by the Alaska Fisheries Science Center (AFSC), of which four (sablefish, Pacific cod, Pacific halibut, and giant grenadier) will be considered in these analyses. The AFSC records number of fish per species collected at each location and calculates a catch per unit effort (CPUE) within each management area from the total number of fish caught divided by the total number of skates, 100‐meter longlines with 45 evenly spaced hooks per line, deployed each day (Sigler & Lunsford, 2009). The CPUE is therefore a standardized measure of catch at each location. Longline surveys recording CPUE have been shown to be an accurate fishery‐independent index of abundance for sablefish (Sigler, 2000) and Pacific halibut (Monnahan & Stewart, 2018) when properly accounting for hook spacing and spatial stratification.

Daily global SST readings, available for dates starting in 1981 through 2012, were obtained from the National Centers for Environmental Information (NOAA, 2015). The data were interpolated and optimized from satellites, buoys, and ships on 1/4° latitude–longitude grids using a method devised by Richard W. Reynolds at the National Centers for Environmental Prediction. A coefficient of variation for SST was derived for each 1/4° latitude–longitude grid for the winter season (November through April), because the groundfish studied in the MESA surveys undergo reproductive activity in the winter months in the waters surrounding Alaska. In addition, evidence has suggested that winter conditions have the greatest influence on groundfish populations (Hollowed & Wooster, 1992). The winter coefficient of variation for SST was calculated as

$$c_{v}=\frac{\sigma }{\mu }$$

at each latitude–longitude pairing, with *σ* being the winter seasonal standard deviation and *μ* the winter season's mean of SST. The coefficient of variation is an improved measure of seasonal SST over the mean, because it standardizes scale and allows us to consider the changes in variation of SST with the changes in mean over time.

Fluctuations in CPUE are likely to be linked to changes across cohorts which are often determined by survival in the first year of life. Water temperature has been found to affect the MESA groundfish covered by my analyses, and juvenile fish are more susceptible to environmental changes than their adult counterparts. Therefore, CPUE for a given year is likely to be linked to the winter SST encountered at the juvenile state by fish entering the adult population. Since the MESA survey targets waters where adults reside during the summer, and the four species covered in my analyses reach maturity at 5–8 years, SST was lagged for years one through five to allow us to capture the effect of SST on the juvenile stages and recruitment. All five lagged SST measures were included for modeling.

I focused on determining the spatio‐temporal catch predictions for four of the species in the MESA study area known as the Gulf of Alaska which ranges from the Dixon Entrance west to Chuginadak Island. The fisheries data were matched with winter SST data from 1982 to 2012. With lagged winter SST included, this created a dataset of CPUE for four groundfish species spanning 23 years from 1990 to 2012. There are 1679 observations each for sablefish, Pacific cod, giant grenadier, and Pacific halibut.

## 4 METHODS

My proposed forecasting method consisted of two parts, a model averaging technique made up of a spatially varying coefficient model with prediction obtained via an ARIMA model and a temporally varying coefficient model with prediction incorporated via an ARIMA model. The proposed method was applied to the Alaska groundfish data. I then compared three main methods of forecasting to my proposed method: a simple ARIMA model with covariates (ARIMAX) with lagged winter SST from 1 to 5 years used as predictors, a naïve spatially varying coefficient model in which the fitted values for the current year were considered the predicted values for the next year, and a hierarchical Bayesian forecasting procedure. The ARIMAX and Bayesian implementations are linear models, while the naïve spatially varying coefficient model and the proposed forecasting method make use of nonlinear models.

The distributions of CPUE values for Pacific cod and Pacific halibut were right‐skewed and were accommodated in the model fitting, while sablefish and giant grenadier CPUE values were Gaussian distributed.

All of the methods were subjected to a leave‐one‐out procedure. This allowed us to determine whether the success of the proposed technique was mainly due to its predictions being verified and adjusted using the leave‐one‐out procedure. Since the naïve spatially varying coefficient model is not a typical forecasting procedure, only the leave‐one‐out setting was considered for this model. For the naïve model, a station was removed from the dataset and the spatial model fitting was performed on the {1, 2, …, *b* − 1, *b* + 1, …, *n*} stations. The spatial forecast obtained after each leave‐one‐out operation is denoted ${\tilde{Y}}_{J}^{\mathrm{sp}(-b)}$, where *b* is the removed station. The ARIMAX and hierarchical Bayesian models go through a similar leave‐one‐out procedure, where a year *c* was removed from the dataset and the ARIMAX (or Bayesian) modeling procedure was performed on the {1, 2, …, *c* − 1, *c* + 1, …, *J* − 1} years. The ARIMAX forecast obtained after each leave‐one‐out procedure is similarly denoted ${\tilde{Y}}_{J}^{t(-c)}$ for the removed year *c*. A mean and standard deviation of the leave‐one‐out forecasts for each station's ARIMAX, Bayesian, and naïve spatial models were calculated. The means of the leave‐one‐out forecasts for each station were used as the final forecast CPUE values for the leave‐one‐out versions of the ARIMAX, Bayesian, and naïve spatial models. Weights for the model averaging of the two component predictions for the proposed method were determined by the standard error of the leave‐one‐out predictions for each. A minimum of 10 observations per location was considered to provide a sufficient number of points to obtain a trend over time; that is, a minimum of 10 yearly observations per station were included in the training datasets that were used to predict the subsequent year. For each of the following models, let *J* be the year for which prediction is sought, where *J* = 2000, 2001, …, 2012.

In Section 4.2.1, I introduce and describe the basic ARIMAX model, the spatially varying coefficient model, and the Bayesian forecasting method to which I compared my proposed forecasting technique. I then show how the ARIMA model and spatially varying coefficient model were combined using model averaging to produce my proposed forecasting method in Section 4.2.2.

### 4.1 Some existing forecasting procedures

#### 4.1.1 ARIMAX model

An ARIMAX of order *p*,* d*,* q* in the form

$$\begin{array}{cc} \left(1-\sum_{m=1}^{p}\phi_{m}L^{m}\right){\left(1-L\right)}^{d}Y_{i} & =\delta +\left(1+\sum_{m=1}^{q}\theta_{m}L^{m}\right)\epsilon_{i}\\ & +\left(1-\sum_{m=1}^{p}\phi_{m}L^{m}\right){\left(1-L\right)}^{d}X_{i}^{T}\beta , \end{array}$$

was fit for each location. The lag operator is denoted by *L*,* ϕ*_*m*_ are the autoregressive parameters, *θ*_*m*_ are the moving average parameters, ***β*** is the predictor coefficient matrix, and ε_*i*_ are the error terms (Box et al., 2013). The predictor vector $X_{i}={\left({\text{SST}}_{i-1},{\text{SST}}_{i-2},{\text{SST}}_{i-3},{\text{SST}}_{i-4},{\text{SST}}_{i-5}\right)}^{T}$ includes lagged winter SST values for one to 5 years. The order (*p*,* d*,* q*) with drift δ/(1 − Σ*ϕ*_*m*_) for the ARIMAX model is automatically determined using minimization of AIC and MLE to determine the best ARIMAX model using the function auto.arima in the forecast package (Hyndman, 2017; Hyndman & Khandakar, 2008) in R (R Core Team, 2017). The right‐skewed distributions of Pacific cod and Pacific halibut CPUEs do not affect the fitting of the ARIMAX models, as each location is fit individually. At most, only one outlier was identified per station for Pacific cod and Pacific halibut when modeling the entire range of training data for one station representative of each of the four management areas (Supporting Information Figures S1 and S2). The fitted ARIMAX model was then used to predict year *J* using known winter SST values from years *J* − 5 to *J* − 1,

$$\begin{array}{cc} \left(1-\sum_{m=1}^{p}{\hat{\phi }}_{m}L^{m}\right){\left(1-L\right)}^{d}{\hat{Y}}_{J} & =\delta +\left(1+\sum_{m=1}^{q}{\hat{\theta }}_{m}L^{m}\right)\epsilon_{J}\\ & +\left(1-\sum_{m=1}^{p}{\hat{\phi }}_{m}L^{m}\right){\left(1-L\right)}^{d}X_{J}^{T}\hat{\beta }, \end{array}$$

with the predicted value for year *J* denoted ${\tilde{Y}}_{J}^{A}$, employing the forecast function from the forecast package.

#### 4.1.2 Naïve spatially varying coefficient model

A spatially varying coefficient model for CPUE of a given species including lagged winter SST for 5 years to one year that varies over space is fit for year *J* − 1,

$$Y_{J-1}=X_{J-1}^{T}G_{J-1}\left(U\right)+\varepsilon_{J-1},$$

where $X_{J-1}={\left(1,{\text{SST}}_{(J-1)-1},{\text{SST}}_{(J-1)-2},{\text{SST}}_{(J-1)-3},{\text{SST}}_{(J-1)-4},{\text{SST}}_{(J-1)-5}\right)}^{T}$ and $G_{\left(J-1\right)}\left(U\right)={\left(g_{0,J-1}\left(U\right),g_{1,J-1}\left(U\right),g_{2,J-1}\left(U\right),g_{3,J-1}\left(U\right),g_{4,J-1}\left(U\right),g_{5,J-1}\left(U\right)\right)}^{T}$ is the functional coefficient vector of winter SSTs with *U* being the longitude‐latitude pairs representing sampled locations along the Gulf of Alaska. The fitted CPUE values for year *J* − 1 given as

$${\hat{Y}}_{J-1}=X_{J-1}^{T}{\hat{G}}_{J-1}\left(U\right)={\hat{g}}_{0(J-1)}+\sum_{k=1}^{5}{\hat{g}}_{k(J-1)}\left(U\right){\text{SST}}_{(J-1)-k}$$

were considered to be the predicted values for year *J*. The predicted values from this naïve spatial model are denoted ${\tilde{Y}}_{J}^{N}$. The spatially varying coefficient models used rank‐based estimation as described in Correia and Abebe (2017). Rank estimation techniques are more suitable than least squares estimation for reducing the influence of outliers and contamination common in fisheries and ecological data on prediction. The rank‐based estimation for varying coefficient models was coded as a modification to the gam function in the mgcv package (Wood, 2006) in R (R Core Team, 2017) and is provided as part of the code included as supplemental material. To accommodate the right‐skewed distributions of Pacific cod and Pacific halibut CPUE values, I used the Gaussian distribution and weights given by the bent score function (Kloke & McKean, 2014) in the varying coefficient model fitting process.

#### 4.1.3 Hierarchical Bayesian forecasting

To implement Bayesian forecasting methods, I chose a hierarchical independent Gaussian process model. Let $Z_{i}$ denote the observed data, and $O_{i}$ be the corresponding true values for station *s*_*r*_, *r* = 1, …, *n* at time *i* = 1, ···, *J* − 1. Also let $Z_{i}={\left(Z\left(s_{1},i\right),\ldots ,Z\left(s_{n},i\right)\right)}^{T}$, $O_{i}={\left(O\left(s_{1},i\right),\ldots ,O\left(s_{n},i\right)\right)}^{T}$, and *N* = *n* × (*J* − 1) be the total number of observations modeled. The Gaussian process model is specified as

$$Z_{i}=O_{i}+\epsilon_{i}\;\text{and}\;O_{i}=X_{i}\beta +\eta_{i}$$

where ***β*** is the regression coefficient vector, and $\epsilon_{i}=\left(\epsilon_{s_{1},i},\ldots ,\epsilon_{s_{n},i}\right)∼N\left(0,\sigma_{\epsilon }^{2}I_{n}\right)$ is the pure error term, $\sigma_{\epsilon }^{2}$ is the unknown variance and **I**_*n*_ is the identity matrix of order *n*. The spatio‐temporal random effects are denoted ***η***_*i*_ = (*η*(*s*_1_, *i*), …, *η*(*s*_*n*_, *i*))^*T*^ ∼ *N*(**0**, *Σ*_*η*_), where $\Sigma_{\eta }=\sigma_{\eta }^{2}S_{\eta }$ is composed of the spatial variance, $\sigma_{\eta }^{2}$, and the spatial correlation matrix, *S*_*η*_. The spatial correlation matrix is derived from the Matérn correlation function

$$\kappa \left(s_{i},s_{j}:\phi ,\nu \right)=\frac{1}{2^{\nu -1}\gamma \left(\nu \right)}(2\sqrt{\nu }\|s_{i}-s_{j}{\left\|\phi \right)}^{\nu }K_{\nu }(2\sqrt{\nu }\|s_{i}-s_{j}\left\|\phi \right),\;\phi >0,\;\nu >0,$$

where *ϕ* controls the correlation decay rate as distance between two spatial points ‖ *s*_*i*_ − *s*_*j*_ ‖  increases, *K*_ν_ is the modified Bessel function of order *ν*, and *ν* controls the smoothness of the random field. Let all of the parameters of the model be denoted $\theta =(\beta ,\sigma_{\epsilon }^{2},\sigma_{\eta }^{2},\phi ,\nu )$, and let *π*(***θ***) denote the prior distributions. The prior distribution for the inverse variance model parameters is given as

$$\left(\frac{1}{\sigma_{\epsilon }^{2}},\frac{1}{\sigma_{\nu }^{2}}\right)∼\Gamma \left(\frac{a}{b},\frac{a}{b^{2}}\right),$$

where *a* = 2 and *b* = 1, while the prior distributions for the mean parameter ***β*** is $N(\mu_{\beta },\delta_{\beta }^{2})$, where *μ*_***β***_ = 0 and $\delta_{\beta }^{2}=10^{10}$. The logarithm of the joint posterior distribution for this Gaussian process model is

$$\begin{array}{cc} \log\pi (\theta ,O,z^{*}|z)\propto & -\frac{N}{2}\log\sigma_{\epsilon }^{2}-\frac{1}{2\sigma_{\epsilon }^{2}}\sum_{i=1}^{J-1}{\left(Z_{i}-O_{i}\right)}^{T}\left(Z_{i}-O_{i}\right)-\frac{1}{2}\log\left|\sigma_{\eta }^{2}S_{\eta }\right|\\ & -\frac{1}{2\sigma_{\eta }^{2}}\sum_{i=1}^{J-1}{\left(O_{i}-X_{i}\beta \right)}^{T}S_{\eta }^{-1}\left(O_{i}-X_{i}\beta \right)+\log\pi \left(\theta \right). \end{array}$$

The Bayesian forecasting method was implemented via the R package spTimer (Bakar & Sahu, 2015).

### 4.2 Spatiotemporally explicit model averaging

The spatiotemporally explicit model averaging (STEMA) technique was derived from the combination of a spatially varying coefficient model (Section 5.3) and a yearly varying coefficient model where latitude–longitude pairs in the spatially varying coefficient model were replaced by year. Each model's fitted values were then used to fit an ARIMA and forecast the year for which prediction was sought. The separate model forecasts were averaged using weights based on standard deviations of the leave‐one‐out procedure, giving more weight to the model with lower standard deviation to produce a final forecast for each station. The components of the STEMA forecasting procedure are described in the following three subsections, with example code of the procedure given in the Supporting Information.

#### 4.2.1 Spatial model with ARIMA

A spatially varying coefficient model as described above was fit over space for each year *i* = 1990, 1991, …, *J* − 1. The fitted CPUE values for year *i* given as

$${\hat{Y}}_{i}^{\mathrm{sp}}=X_{i}^{T}{\hat{G}}_{i}\left(U\right)={\hat{g}}_{0i}+\sum_{k=1}^{5}{\hat{g}}_{\mathrm{ki}}\left(U\right){\text{SST}}_{i-k}$$

were then used to fit an ARIMA model for each station,

$$\left(1-\sum_{m=1}^{p}\phi_{m}L^{m}\right){\left(1-L\right)}^{d}{\hat{Y}}_{i}^{\mathrm{sp}}=\delta +\left(1+\sum_{m=1}^{q}\theta_{m}L^{m}\right)\epsilon_{i}$$

yielding the fitted ARIMA values ${\tilde{Y}}_{J}^{\mathrm{sp}}$, where the order (*p*, *d*, *q*) with drift δ/(1 − *Σϕ*_*m*_) is automatically determined using the auto.arima function as described in Section 4.1.1. The two‐step process allows for inclusion of multiple lagged winter SST variables smoothed over space in the varying coefficient model setting while providing a method for future prediction which is not available in these models.

#### 4.2.2 Temporal model with ARIMA

A time varying coefficient model of the form

$$Y_{J-1}=g_{0J}\left(t\right)+g_{1J}\left(t\right){\text{SST}}_{t-5}+\varepsilon_{J-1}$$

was fit for each location, where *i* = 1990, 1991, …, *J* − 1; *t* = 1990, 1991, …, *J* − 1; and SST_*t*‐5_ is the winter SST for that location lagged by five years. The fitted values from this model,

$${\hat{Y}}_{i}^{t}={\hat{g}}_{0J}\left(i\right)+{\hat{g}}_{1J}\left(i\right){\text{SST}}_{i-5},$$

were then used to fit an ARIMA model

$$\left(1-\sum_{m=1}^{p}\phi_{m}L^{m}\right){\left(1-L\right)}^{d}{\hat{Y}}_{i}^{t}=\delta +\left(1+\sum_{m=1}^{q}\theta_{m}L^{m}\right)\epsilon_{i},$$

yielding the fitted ARIMA values ${\tilde{Y}}_{J}^{t}$, where the order (*p*, *d*, *q*) with drift δ/(1 − *Σϕ*_*m*_) is automatically determined as described for the spatial model. The coefficient model smooths the CPUEs for each location, thereby allowing the time series model to determine a more accurate trend despite highly variable CPUE values.

#### 4.2.3 Model averaging

The STEMA method underwent the same leave‐one‐out procedures as described for the ARIMA and naïve spatial models, where the temporal model with ARIMA used the temporal leave‐one‐out procedure and the naïve spatial model utilized the spatial leave‐one‐out steps. For the STEMA technique, the means of the spatial and temporal leave‐one‐out procedures ($mean({\tilde{Y}}_{J}^{\mathrm{sp}})$ and $mean({\tilde{Y}}_{J}^{t})$, respectively) were weighted for each location using a ratio of the spatial ($\sigma_{sp_{n}}$) and temporal ($\sigma_{t_{n}}$) standard deviations from the leave‐one‐out predictions,

$$\omega_{\mathrm{sp}}=\frac{\sigma_{t}}{\sigma_{\mathrm{sp}}+\sigma_{t}}\;\text{and}\;\omega_{t}=\frac{\sigma_{\mathrm{sp}}}{\sigma_{\mathrm{sp}}+\sigma_{t}},$$

where *ω*_*sp*_ + *ω*_*t*_ = 1. The final spatiotemporally explicit model averaged prediction was obtained for each location by

$${\tilde{Y}}_{J}=\omega_{\mathrm{sp}}\;mean\left({\tilde{Y}}_{J}^{\mathrm{sp}}\right)+\omega_{t}\;mean\left({\tilde{Y}}_{J}^{t}\right).$$

The standard error of the spatiotemporally explicit model averaged predictions is given as

$$\mathrm{SE}\left({\tilde{Y}}_{J}\right)=\sqrt{\omega_{\mathrm{sp}}^{2}\;\mathrm{SEM}\;\left({\tilde{Y}}_{J}^{\mathrm{sp}}\right)+\omega_{t}^{2}\;\mathrm{SEM}\;\left({\tilde{Y}}_{J}^{t}\right)},$$

where SEM is the standard error of the mean.

## 5 ASSESSMENT OF FORECAST PERFORMANCE VIA CROSS‐VALIDATION

### 5.1 Model comparison

A time series cross‐validation based on one‐step forecasts was performed on the ARIMAX model (A), the hierarchical Bayesian model (B), the naïve spatially varying coefficient model (N^1^), the STEMA technique, and the leave‐out‐out versions of the ARIMAX (A^1^) and Bayesian (B^1^) models. I consider *h* to be the minimum number of years needed to create a reliable forecast and proceed as follows: for *f* = 1, 2, …, *T* − *h*, where *T* is the total number of years available and *j* = *h* + *f*, train on *F*_*h*_, …, *F*_*j*‐1_, and forecast and validate on *F*_*j*_. For each estimation technique, a forecast ${\hat{Y}}_{\mathrm{aj}}$, $a=A,\;A^{1},\;B,\;B^{1},\;N^{1},\;\text{STEMA}$ was computed from the training sets, and the error on the validation set was recorded as

$$e_{\mathrm{aj}}=Y_{j}-{\hat{Y}}_{\mathrm{aj}}.$$

To compare estimation techniques, I used a Friedman rank sum test on the absolute errors to determine whether there were significant differences among methods (Friedman, 1937). If the Friedman test indicated significant differences, I then performed pairwise multiple comparisons on the differences between the absolute errors for each pair of methods (Bretz, Westfall, & Hothorn, 2016; Tukey, 1949). In order to control for the effect of location, a generalized linear mixed model was fit with the stations set as random effects. *p*‐Values calculated for the pairwise tests were adjusted using the Benjamini–Hochberg procedure to control the false discovery rate (Benjamini & Hochberg, 1995). If the difference was significantly less than zero, the first of the two compared methods was the method that produced smaller errors; if the difference was significantly greater than zero, the second method produced smaller errors. The mixed model was fit using the glmer function in the lme4 package (Bates, Mächler, Bolker, & Walker, 2015), while the pairwise multiple comparisons were performed in the multcomp package (Hothorn, Bretz, & Westfall, 2008) using the glht function in R (R Core Team, 2017).

### 5.2 Forecast performance in the presence of an environmental covariate

In order to determine whether adding SST to the models improved forecasting, a null model for each of the four techniques was fit, subjected to the same leave‐one‐out procedure as described previously, and a forecast obtained for each. The null A and A^1^ models are of the form

$$\left(1-\sum_{m=1}^{p}\phi_{m}L^{m}\right){\left(1-L\right)}^{d}{\hat{Y}}_{i}=\delta +\left(1+\sum_{m=1}^{q}\theta_{m}L^{m}\right)\epsilon_{i},$$

where the order *p*, *d*, *q* is determined as before.

The fitted CPUE values for year *J* − 1 from the null naïve spatially varying coefficient model given as

$${\hat{Y}}_{J-1}={\hat{G}}_{J-1}\left(U\right)={\hat{g}}_{0(J-1)}$$

are considered to be the predicted values for year *J*.

The spatiotemporally model averaged forecasts were derived from the two null varying coefficient models

$$Y_{i}^{sp_{0}}=g_{0i}+\varepsilon_{i}\;\text{and}\;Y_{J-1}^{t_{0}}=g_{0J}\left(t\right)+\varepsilon_{J-1},$$

of which the fitted values were each used to fit ARIMA models following the steps in Section 4.2. The forecasts obtained from those fitted ARIMA models were averaged as described in Section 4.2.3 to form the final prediction. The null model errors were compared to the errors of their model counterparts which include winter SST for each method using one‐sided Wilcoxon signed‐rank tests, where the errors are matched by station and year. If SST is important to forecasting, the inclusion of SST in the model will significantly reduce forecasting error.

In order to obtain the magnitude of the effect of winter SST on prediction using the preferred methods, I calculated a rank correlation *r* statistic using the asymptotic normal distribution of the Wilcoxon signed‐rank statistic *W* on the absolute error differences *D*_*i*_ between the null model and the SST model. *W* is calculated as

$$W=\sum_{i=1}^{N}\text{Rank}(|D_{i}\left|\right)\times I\left(D_{i}>0\right),$$

where *N* is the total number of calculated errors. Under the hypothesis that winter SST has no impact on prediction, *W* is asymptotically normal as

$$Z=\frac{W-\frac{N(N+1)}{4}}{\sqrt{\frac{N(N+1)(2N+1)}{24}}}$$

(Hollander & Wolfe, 1999). The rank correlation is given by

$$r=\frac{Z}{\sqrt{N}}$$

with estimated variance (1 − *r* ^2^)/(*N* − 2) (Rosenthal, Cooper, & Hedges, 1994). Small, medium, and large effect sizes are .10, .30, and .50, respectively (Cohen, 1992).

### 5.3 Control of Bayesian parameter *ϕ* in cross‐validation

One issue that arises from using the time series cross‐validation on the Bayesian forecasting method is the fluctuation in spatial point acceptance rate as the available years of data change. While the spatial decay parameter *ϕ* can be chosen by the user to obtain optimal acceptance rate of spatial points for the calculation of the spatial correlation matrix, the appropriate value of *ϕ* changes given varying data structure. There is also insufficient guidance on how forecast values are affected by misspecification of *ϕ*. According to Bakar and Sahu (2015), the choice of *ϕ* is obtained with acceptance rates between 20% and 40%, which is justified by Gelman, Carlin, Stern, and Rubin (2004).

I chose to apply a search similar to Paci, Gelfand, and Holland (2013) for the optimal *ϕ* value by fitting the current dataset with values of *ϕ* starting at 10 and decreasing by an order of magnitude of 1 for each subsequent fitting. Once the model achieved an acceptance rate closest to 32%, that model was then used to obtain forecasting estimates for year *J*. This ensured that *ϕ* was selected for each model fitting step to always obtain an optimal acceptance rate despite the changing size of training data. Variable training data that occur when using the temporal cross‐validation affect the spatial information available, making a fixed value of *ϕ* unsuitable for accurate forecasting using the Bayesian method with temporal cross‐validation.

## 6 RESULTS

Friedman tests for all four species revealed significant differences across model techniques (sablefish: *χ* ^2^ = 74.022, *p *≤ 0.0001; Pacific cod: *χ* ^2^ = 365.501, *p *≤ 0.0001; Pacific halibut: *χ* ^2^ = 152.471, *p *≤ 0.0001; giant grenadier: *χ* ^2^ = 460.030, *p *≤ 0.0001). The STEMA method had lowest mean absolute errors for Gaussian distributed species (sablefish and giant grenadier) when ignoring station and year effects (Table 1). Pairwise multiple comparisons were therefore performed on method pairings for all species to determine the best methods of forecasting for each species. The results of these pairwise comparison tests of the different methods are given in Tables 2, 3, 4, 5. Based on the differences in mean absolute errors that are significant, the following methods had the lowest significant absolute errors: the STEMA method for sablefish, the naïve and STEMA methods for Pacific cod, the naïve method for Pacific halibut, and the STEMA method for giant grenadier. STEMA did not significantly improve forecasting over the N^1^ model in the case of Pacific cod, and the N^1^ method outperformed STEMA in lowering forecasting errors for Pacific halibut.

**Table 1:** Mean absolute error of each method for four species, ignoring station effects

	A	A^1^	N^1^	B	B^1^	STEMA
Sablefish	1.771	1.688	1.612	1.558	1.562	**1.369**
Pacific cod	0.280	0.267	**0.136**	0.180	0.175	0.145
Pacific halibut	0.293	0.285	**0.199**	0.205	0.204	0.205
Giant grenadier	1.599	1.480	1.614	1.844	1.839	**1.086**

*Note*

Lowest mean absolute errors for each species are in bold.

**Table 2:** Pairwise multiple comparisons of absolute errors of forecasting methods with winter SST included in the models for *sablefish*

Linear hypotheses	Estimate	Std. error	*z* value	Pr(>|*z*|)
A^1^–A	−0.050	0.038	−1.323	0.253
B–A	−0.130	0.038	−3.435	**0.002**
B–A^1^	−0.080	0.038	−2.115	0.057
B^1^–A	−0.128	0.038	−3.369	**0.002**
B^1^–A^1^	−0.078	0.038	−2.049	0.061
B^1^–B	0.002	0.038	0.066	0.947
N^1^–A	−0.090	0.038	−2.378	**0.033**
N^1^–A^1^	−0.040	0.038	−1.057	0.335
N^1^–B	0.040	0.038	1.058	0.335
N^1^–B^1^	0.038	0.038	0.993	0.344
STEMA–A	−0.254	0.038	−6.707	**0.000**
STEMA–A^1^	−0.204	0.038	−5.386	**0.000**
STEMA–B	−0.124	0.038	−3.264	**0.002**
STEMA–B^1^	−0.126	0.038	−3.331	**0.002**
STEMA–N^1^	−0.164	0.038	−4.330	**0.000**

*Notes*.

*p*‐Values are adjusted using false discovery rate method. A *p*‐value < 0.05 indicates the difference in absolute errors of the comparison is significant (in bold). Differences significantly less than zero indicate the first of the two compared methods was the method that produced smaller errors; estimates significantly greater than zero indicate the second method produced smaller errors.

1 Leave‐one‐out procedure used.

**Table 3:** Pairwise multiple comparisons of absolute errors of forecasting methods with winter SST included in the models for *Pacific cod*

Linear hypotheses	Estimate	Std. error	*z* value	Pr(>|*z*|)
A^1^–A	−0.033	0.040	−0.835	0.454
B–A	−0.275	0.040	−6.828	**0.000**
B–A^1^	−0.241	0.040	−6.004	**0.000**
B^1^–A	−0.307	0.040	−7.626	**0.000**
B^1^–A^1^	−0.273	0.040	−6.802	**0.000**
B^1^–B	−0.032	0.040	−0.800	0.454
N^1^–A	−0.525	0.040	−12.996	**0.000**
N^1^–A^1^	−0.492	0.040	−12.185	**0.000**
N^1^–B	−0.251	0.040	−6.226	**0.000**
N^1^–B^1^	−0.219	0.040	−5.434	**0.000**
STEMA–A	−0.550	0.040	−13.699	**0.000**
STEMA–A^1^	−0.516	0.040	−12.879	**0.000**
STEMA–B	−0.275	0.040	−6.855	**0.000**
STEMA–B^1^	−0.243	0.040	−6.061	**0.000**
STEMA–N^1^	−0.024	0.040	−0.609	0.542

*Notes*.

*p*‐Values are adjusted using false discovery rate method. A *p*‐value < 0.05 indicates the difference in absolute errors of the comparison is significant (in bold). Differences significantly less than zero indicate the first of the two compared methods was the method that produced smaller errors; differences significantly greater than zero indicate the second method produced smaller errors.

1 Leave‐one‐out procedure used.

**Table 4:** Pairwise multiple comparisons for absolute errors of forecasting methods with winter SST included in the models for *Pacific halibut*

Linear hypotheses	Estimate	Std. error	*z* value	Pr(>|*z*|)
A^1^–A	−0.034	0.006	−5.413	**0.000**
B–A	−0.384	0.006	−61.003	**0.000**
B–A^1^	−0.350	0.009	−39.580	**0.000**
B^1^–A	−0.387	0.006	−61.442	**0.000**
B^1^–A^1^	−0.353	0.009	−39.895	**0.000**
B^1^–B	−0.003	0.009	−0.334	0.739
N^1^–A	−0.423	0.006	−67.557	**0.000**
N^1^–A^1^	−0.389	0.009	−44.229	**0.000**
N^1^–B	−0.040	0.009	−4.494	**0.000**
N^1^–B^1^	−0.037	0.009	−4.160	**0.000**
STEMA–A	−0.391	0.006	−62.332	**0.000**
STEMA–A^1^	−0.357	0.009	−40.516	**0.000**
STEMA–B	−0.008	0.009	−0.860	0.450
STEMA–B^1^	−0.005	0.009	−0.526	0.642
STEMA–N^1^	0.032	0.009	3.634	**0.000**

*Notes*.

*p*‐Values are adjusted using false discovery rate method. A *p*‐value < 0.05 indicates the difference in absolute errors of the comparison is significant (in bold). Differences significantly less than zero indicate the first of the two compared methods was the method that produced smaller errors; differences significantly greater than zero indicate the second method produced smaller errors.

1 Leave‐one‐out procedure used.

**Table 5:** Pairwise multiple comparisons for absolute errors of forecasting methods with winter SST included in the models for *giant grenadier*

Linear hypotheses	Estimate	Std. error	*z* value	Pr(>|*z*|)
A^1^–A	−0.075	0.040	−1.887	0.068
B–A	0.274	0.040	6.797	**0.000**
B–A^1^	0.349	0.040	8.661	**0.000**
B^1^–A	0.271	0.040	6.733	**0.000**
B^1^–A^1^	0.346	0.040	8.595	**0.000**
B^1^–B	−0.002	0.040	−0.058	0.954
N^1^–A	0.010	0.040	0.255	0.856
N^1^–A^1^	0.085	0.040	2.134	**0.041**
N^1^–B	−0.263	0.041	−6.500	**0.000**
N^1^–B^1^	−0.261	0.041	−6.435	**0.000**
STEMA–A	−0.367	0.040	−9.218	**0.000**
STEMA–A^1^	−0.292	0.040	−7.333	**0.000**
STEMA–B	−0.641	0.040	−15.895	**0.000**
STEMA–B^1^	−0.639	0.040	−15.817	**0.000**
STEMA–N^1^	−0.378	0.040	−9.478	**0.000**

*Notes*.*p*‐Values are adjusted using false discovery rate method. A *p*‐value < 0.05 indicates the difference in absolute errors of the comparison is significant (in bold). Differences significantly less than zero indicate the first of the two compared methods was the method that produced smaller errors; differences significantly greater than zero indicate the second method produced smaller errors.

1 Leave‐one‐out procedure used.

The results of the one‐sided Wilcoxon signed‐rank tests for comparing models including winter SST to those without for all forecasting methods are summarized in Table 6. For all four groundfish species, the STEMA method of forecasting had significantly reduced absolute errors when lagged winter SSTs were included as covariates. For Pacific cod, Pacific halibut, and giant grenadier, the models using the naïve method of forecasting benefited significantly from the addition of SST. The A and A^1^ methods for all species had higher absolute errors upon the addition of SST to the models.

**Table 6:** One‐sided Wilcoxon signed‐rank test comparing the absolute errors of the rank‐estimated GAMs including winter SST with the absolute errors of the null model

Species	Method	Abs. errors w/o SST	Abs. errors w/SST	*p*‐Value
Sablefish	A	1.455 (1.017)	1.771 (1.291)	1.000
	A^1^	1.396 (1.055)	1.688 (1.237)	1.000
	B	1.626 (1.053)	1.558 (1.034)	0.000
	B^1^	1.619 (1.059)	1.562 (1.046)	0.000
	N^1^	1.585 (1.186)	1.612 (1.161)	0.845
	**STEMA**	**1.386 (1.057)**	**1.369 (1.093)**	**0.006**
Pacific cod	A	0.157 (0.180)	0.280 (0.314)	1.000
	A^1^	0.165 (0.186)	0.267 (0.289)	1.000
	B	0.222 (0.146)	0.180 (0.120)	0.000
	B^1^	0.215 (0.138)	0.175 (0.116)	0.000
	**N** 1	**0.142 (0.136)**	**0.136 (0.134)**	**0.000**
	**STEMA**	**0.153 (0.159)**	**0.145 (0.162)**	**0.000**
Pacific halibut	A	0.242 (0.153)	0.293 (0.286)	1.000
	A^1^	0.227 (0.147)	0.285 (0.277)	1.000
	B	0.193 (0.177)	0.205 (0.176)	1.000
	B^1^	0.193 (0.177)	0.204 (0.176)	1.000
	**N** 1	**0.207 (0.181)**	**0.199 (0.179)**	**0.001**
	STEMA	0.208 (0.165)	0.205 (0.164)	0.011
Giant grenadier	A	1.041 (1.126)	1.599 (1.505)	1.000
	A^1^	1.055 (1.131)	1.480 (1.393)	1.000
	B	1.847 (1.381)	1.844 (1.572)	0.020
	B^1^	1.817 (1.391)	1.839 (1.590)	0.168
	N^1^	1.747 (1.474)	1.614 (1.386)	0.001
	**STEMA**	**1.149 (1.165)**	**1.086 (1.109)**	**0.000**

Notes.

Mean absolute errors and standard deviations in parentheses are given. A *p*‐value < 0.05 indicates the absolute errors of the models including winter SST are significantly smaller than the absolute errors of the null models. Methods with lowest forecast errors as determined by the pairwise multiple comparisons in Tables 2, 3, 4, 5 are in bold.

1 Leave‐one‐out procedure used.

## 7 DISCUSSION

I propose a model averaging forecasting technique to capture both spatial and temporal information in ecological time series data. The method incorporates a flexible model capable of handling spatially dependent covariates with the familiarity and forecasting ability of ARIMA models for time series analysis. I applied the proposed method to catch data of four ecologically and commercially important species of groundfish where information regarding juvenile survival is often difficult to obtain and life‐history data are sparse or unknown, thereby making projections of population and catch challenging.

The A and A^1^ models were inadequate for forecasting annual catch by location for any of the four species in the analysis. Previous studies indicated that ARIMA models outperform other linear time series methods when forecasting monthly data (Stergiou, Christou, & Petrakis, 1997); however, ARIMA is less suited to yearly data (Stergiou & Christou, 1996) and nonlinear time series (Koutroumanidis et al., 2006). Spatial information is therefore an important component of modeling and forecasting catch in mobile marine species. A more flexible model, such as the varying coefficient model I employed, is also more desirable for capturing unknown nonlinear relationships between the response and predictors in complex systems.

The proposed STEMA method was always chosen as a preferred method for forecasting over ARIMAX and Bayesian models. It should be noted that catches for the two species in which STEMA did not significantly outperform the N^1^ model were right‐skewed, as noted in Section 4. For these two species, the N^1^ and STEMA methods which employed the rank‐based estimation of Correia and Abebe (2017) using a Gaussian distribution with bent score function outperformed the A, A^1^, B, and B^1^ techniques for forecasting. Correia and Abebe (2017) showed that a bent score function in the estimation of generalized additive models (GAMs) improved model fit for Pacific cod catch over modeling with a Gamma distribution using a log link function. This Gamma distribution is one of the typical methods employed in fisheries research to deal with skewed catch data. However, the bent score function more appropriately accounted for skewness in the distribution of Pacific cod catch. The lower absolute forecasting errors for models using rank‐based estimation (N^1^ and STEMA) for Pacific cod and Pacific halibut data indicate that the success of the bent score function to accommodate skewness also reaches to forecasting applications of varying coefficient models, which are an extension of GAMs. The application of the estimation techniques of Correia and Abebe (2017) to the varying coefficient models used in STEMA takes advantage of the improved fit for heavy‐tailed distributions common in fisheries data.

While the STEMA method did not beat the N^1^ method in two of the species, naïve methods are notoriously difficult to beat in time series forecasting, particularly for annual data (Athanasopoulos, Hyndman, Song, & Wu, 2011; Kilian & Taylor, 2003). The bent score function used in the estimation of the varying coefficient models in the N^1^ and STEMA methods for Pacific cod and Pacific halibut reduces the effect of extreme values on estimation. This dampens large deviations in Pacific cod and Pacific halibut CPUE and produces fitted values closer to the mean CPUE. Naïve methods will invariably do better for very short‐term forecasts, because responses close to their mean values behave more like a random walk (Kilian & Taylor, 2003). The fact that STEMA was better than or equal to the N^1^ method for short‐term forecasts in three out of the four species despite the known strengths of the naïve method illustrates the effectiveness of the STEMA method.

A statistically significant reduction in forecasting errors was discernible when winter SST was included for all preferred forecasting methods with lowest absolute errors. Thus, adding covariates relevant to the ecology of the species under consideration can significantly improve the forecasting power of a model. The inclusion of SST in the A and A^1^ models increases the absolute forecasting errors for all species. Covariates in the ARIMAX and Bayesian settings are incorporated linearly; however, the effect of winter SST on groundfish catch is likely to be nonlinear (Laurel, Hurst, Copeman, & Davis, 2008; Rooper & Martin, 2009; Sadorus, Mantua, Essington, Hickey, & Hare, 2014), which is apparent in Supporting Information Figure S3; therefore, the effect's nonlinear shape is not being taken into account in the A, A^1^, B, and B^1^ forecasting methods.

**Figure 1:**
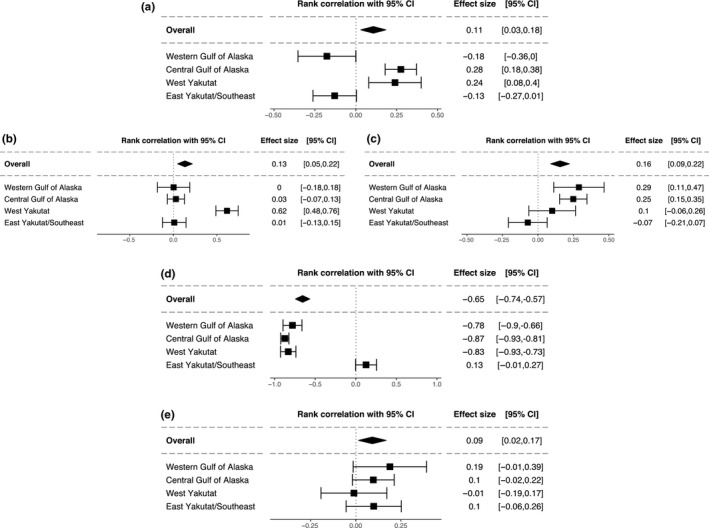
Effect size of lagged winter SST on CPUE of each species broken down by management area using best forecasting method as determined by the results of pairwise multiple comparisons: (a) Sablefish (STEMA), (b) Pacific cod (N^1^), (c) Pacific cod (STEMA), (d) Pacific halibut (N^1^), and (e) Giant grenadier (STEMA). The rank correlation *r* statistic is given as the effect size

I broke down size of the effect of winter SST on groundfish catch for the preferred forecasting methods by management area as defined by the AFSC for the MESA survey in Figure 1, where the order of the areas is from west to east along the coast of Alaska. Effect size of winter SST on the forecasting errors varies from none to large as defined by Cohen (1992), depending upon species and management area. It is likely that a given species will respond to SST differently in different locations (Rouyer, Fromentin, Hidalgo, & Stenseth, 2014), which is evident by the variable effect sizes of winter SST by station provided in Supporting Information Figures [Link], [Link], [Link], [Link], [Link]. Other factors such as habitat, prey availability, and proximity to other individuals of the same species may influence the effects of SST on survival of juveniles. For example, while all these groundfish are not schooling species, Stoner and Ottmar (2004) found that young Pacific halibut were more likely to locate and attack baits in groups than when solitary. Therefore, Pacific halibut, which experience reduced ability to locate bait in low temperatures, may instead successfully find bait in the presence of other individuals. SST may also be a proxy for other environmental variables, such as oxygen levels, ocean mixing, and plankton availability, that may affect these groundfish to varying degrees. Sadorus et al. (2014) found a significant relationship between dissolved oxygen and catch rates of Pacific halibut. Primary production (plankton) concentration and distribution and subsequent changes in secondary production levels have also been linked to groundfish abundance (Francis et al., 1998; McGowan, Cayan, & Dorman, 1998). Correia and Abebe (2017) found improved prediction when adding winter SST to models for sablefish and Pacific cod catches; however, model fit did not substantially improve with the addition of winter SST. Therefore, the link of SST to groundfish catches is likely complex and difficult to quantify directly in wild populations.

I have shown that spatial information is crucial to forecasting in large‐scale data, and my STEMA technique is successful in reducing forecasting errors. Additionally, the inclusion of environmental covariates can improve forecasting in many cases. As is the case with forecasting and prediction techniques, predictions outside the range of observed covariates (i.e., extrapolation) are ill‐advised (Conn, Johnson, & Boveng, 2015; Steyerberg et al., 2010). Forecasts more than one time point ahead can be achieved for the STEMA technique via the forecast function after fitting the ARIMA models in the spatial model with ARIMA (Section 7) and temporal model with ARIMA (Section ACKNOWLEDGMENT). The leave‐one‐out procedure and model averaging would be performed as described (Section AUTHOR CONTRIBUTION) for each time point for which forecasts were estimated. While the proposed technique is only suitable to forecast future, regular time points for the same locations, this is typically desirable for many ecological and epidemiological analyses where predicting the status of a fixed population at future time points is desired. It would be feasible to extend STEMA‐generated forecasts to new locations by using any of several spatial interpolation methods including inverse distance weighting, kriging, and smoothing splines. Migratory and irregular population values can also be forecast provided seasonality is appropriately accounted for in the ARIMA model structure portion of the STEMA method. The STEMA technique is also as intuitive, accessible, and simpler to deploy than other forecasting methods compared in this paper, making it a suitable forecasting method for population ecology, fisheries and wildlife management, vector‐borne disease research and monitoring, and econometrics.

## AUTHOR CONTRIBUTION

Hannah Correia, as the sole writer of the manuscript, contributed wholly to the conception, development, analysis, and interpretation of the work.

## DATA ACCESSIBILITY

This study was an analysis of existing data that are publicly available from the NOAA at https://noaa-fisheries-afsc.data.socrata.com/dataset/AFSC-ABL-Longline-Sablefish-Survey/itxd-qjvg and https://www.ncei.noaa.gov/data/sea-surface-temperature-optimum-interpolation/access/avhrr-only/ and were brought together for this analysis. The compiled data supporting this research are openly available from the Dryad data archive at https://doi.org/10.5061/dryad.s23g7bc.
