# Supplementary figures and images for: Spatiotemporally explicit model averaging for forecasting of Alaskan groundfish catch
Source: Ecol Evol. 2018 Dec 7;8(24):12308–21. doi: 10.1002/ece3.4488 (PMC6308877; doi:10.1002/ece3.4488)

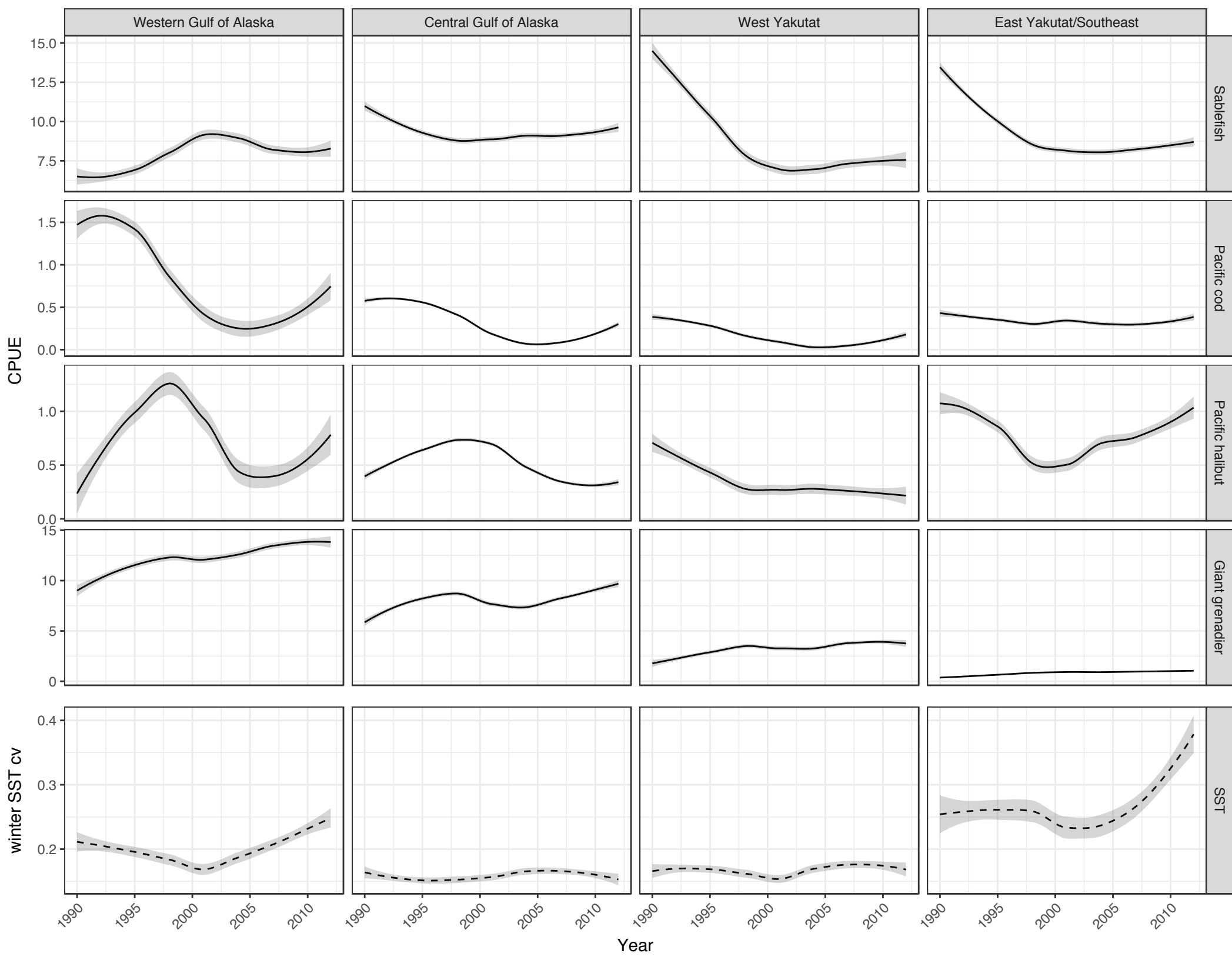

Supplement: Supplementary file 3 [file ECE3-8-12308-s003.pdf]

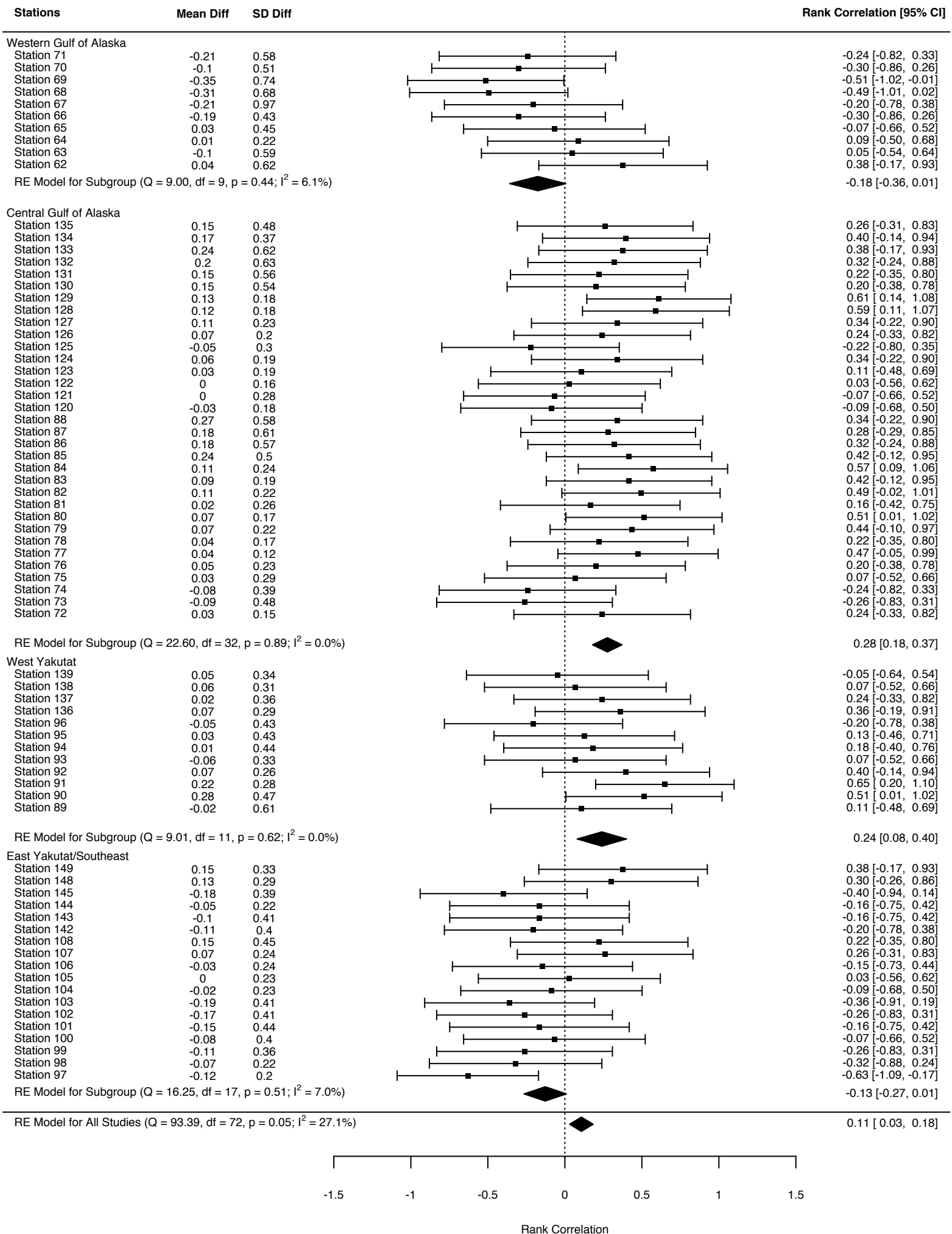

Supplement: Supplementary file 4 [file ECE3-8-12308-s004.pdf]

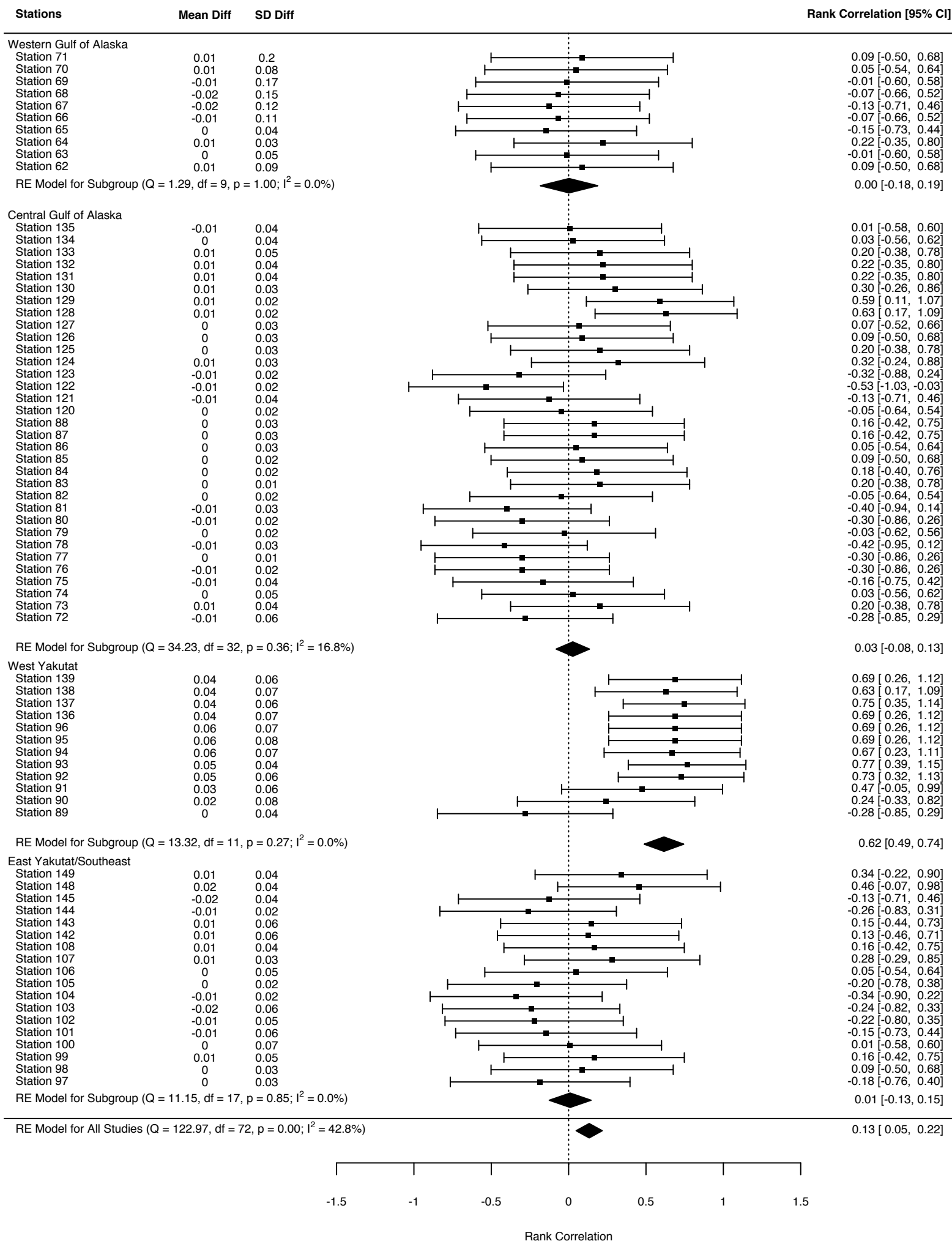

Supplement: Supplementary file 5 [file ECE3-8-12308-s005.pdf]

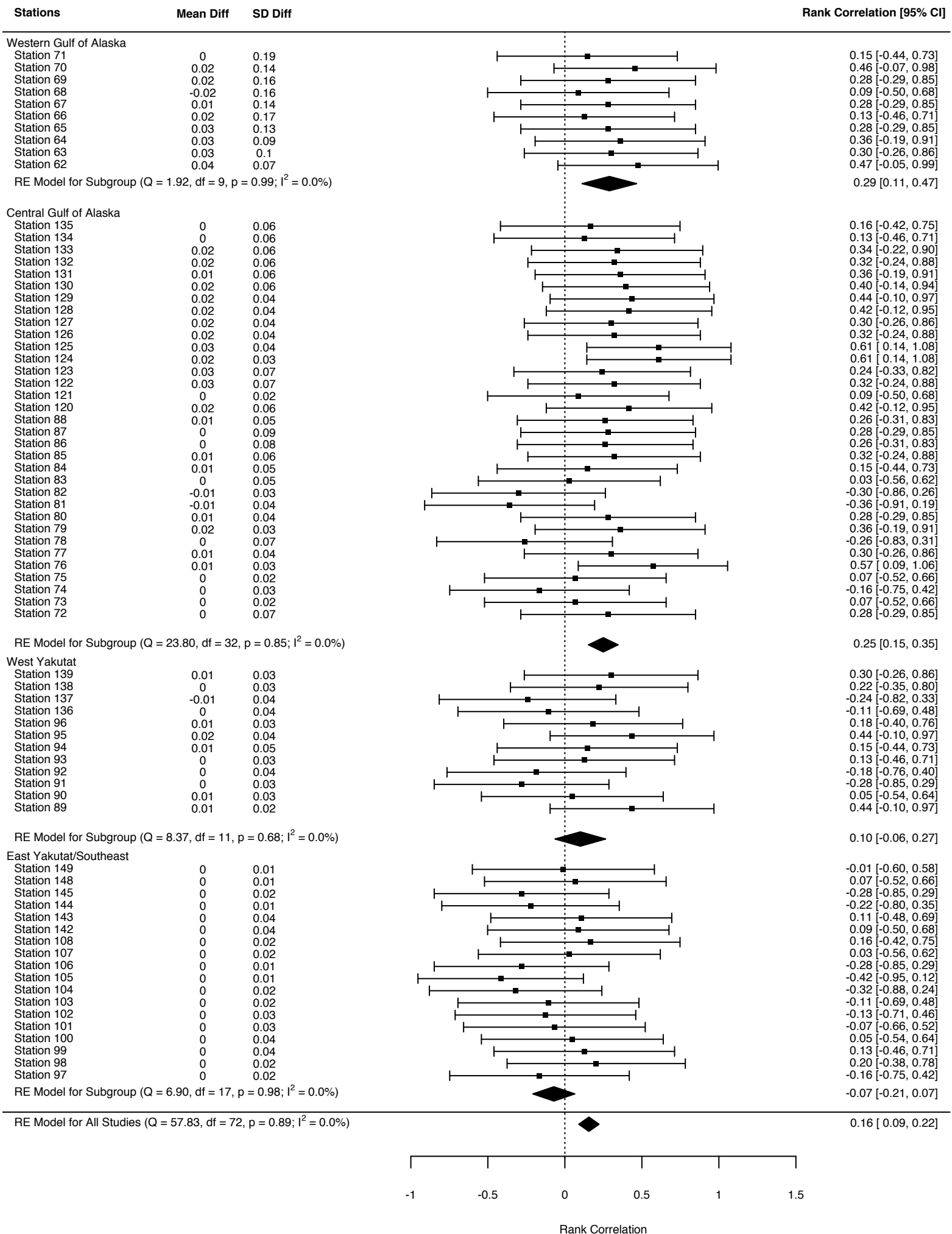

Supplement: Supplementary file 6 [file ECE3-8-12308-s006.pdf]

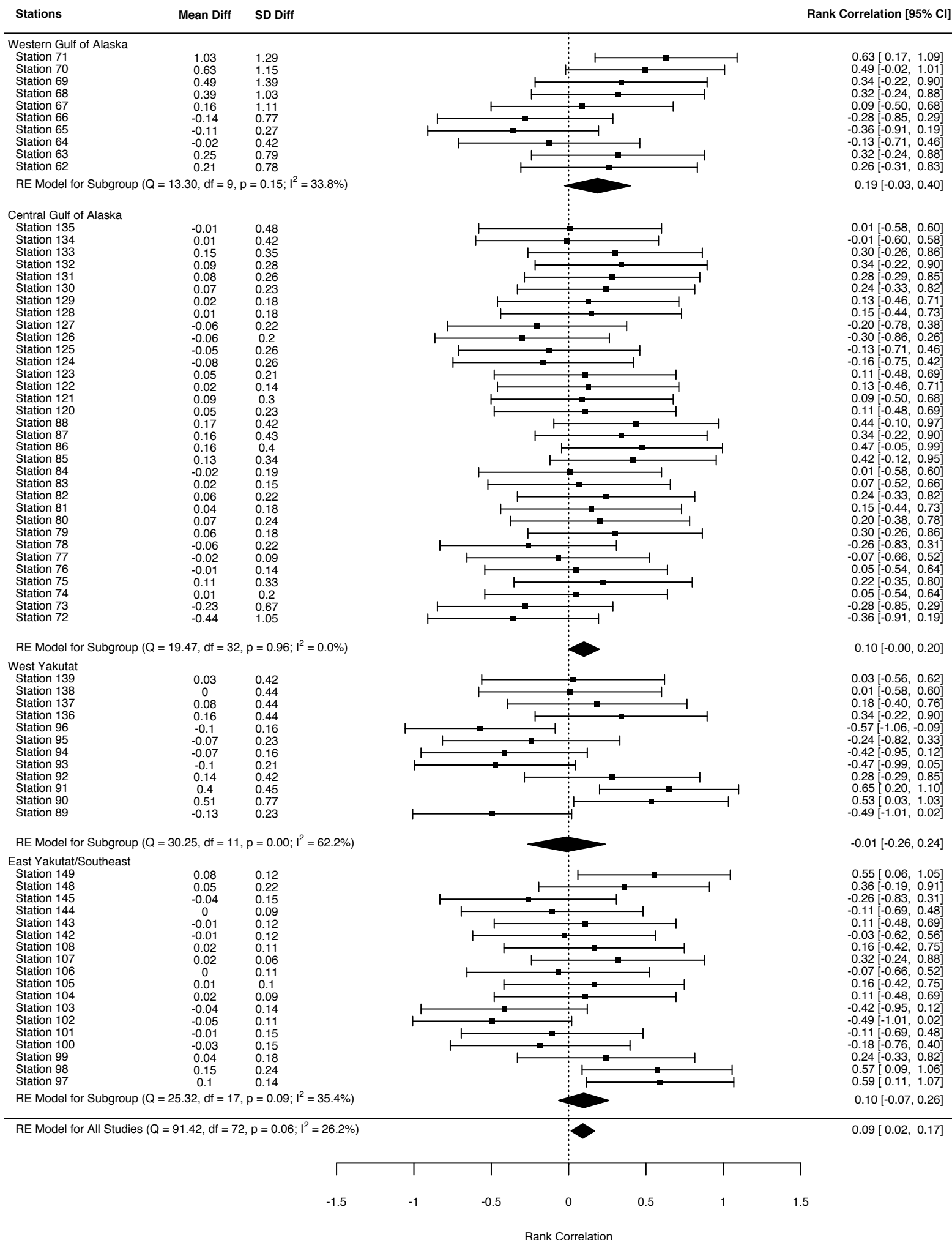

Supplement: Supplementary file 8 [file ECE3-8-12308-s008.pdf]
