# Supplementary material for: Spatiotemporally explicit model averaging for forecasting of Alaskan groundfish catch
Source: Ecol Evol. 2018 Dec 7;8(24):12308–21. doi: 10.1002/ece3.4488 (PMC6308877; doi:10.1002/ece3.4488)

Stations	Mean Diff	SD Diff	Rank Correlation [95% CI]
**Western Gulf of Alaska**			
Station 71	-1.36	1.57	-0.75 [-1.14, -0.35]
Station 70	-1.95	1.45	-0.88 [-1.16, -0.60]
Station 69	-1.26	1.3	-0.79 [-1.15, -0.42]
Station 68	-1.33	1.38	-0.80 [-1.16, -0.45]
Station 67	-1.09	1.3	-0.73 [-1.13, -0.32]
Station 66	-1.23	1.3	-0.80 [-1.16, -0.45]
Station 65	-1.18	1.3	-0.73 [-1.13, -0.32]
Station 64	-1.33	1.33	-0.80 [-1.16, -0.45]
Station 63	-1.13	1.28	-0.71 [-1.13, -0.29]
Station 62	-1.14	1.39	-0.63 [-1.09, -0.17]
RE Model for Subgroup (Q = 1.26, df = 9, p = 1.00; I^2 = 0.0%)			-0.78 [-0.90, -0.66]
**Central Gulf of Alaska**			
Station 135	-2.38	1.3	-0.88 [-1.16, -0.60]
Station 134	-2.33	1.47	-0.88 [-1.16, -0.60]
Station 133	-2.2	1.26	-0.88 [-1.16, -0.60]
Station 132	-2.22	1.31	-0.88 [-1.16, -0.60]
Station 131	-2.23	1.24	-0.88 [-1.16, -0.60]
Station 130	-2.24	1.24	-0.88 [-1.16, -0.60]
Station 129	-2.27	1.26	-0.88 [-1.16, -0.60]
Station 128	-2.28	1.27	-0.88 [-1.16, -0.60]
Station 127	-2.25	1.27	-0.88 [-1.16, -0.60]
Station 126	-2.26	1.28	-0.88 [-1.16, -0.60]
Station 125	-2.27	1.27	-0.88 [-1.16, -0.60]
Station 124	-2.33	1.36	-0.88 [-1.16, -0.60]
Station 123	-2.26	1.29	-0.88 [-1.16, -0.60]
Station 122	-2.23	1.3	-0.86 [-1.16, -0.56]
Station 121	-2.2	1.3	-0.86 [-1.16, -0.56]
Station 120	-2.19	1.31	-0.86 [-1.16, -0.56]
Station 88	-2.15	1.33	-0.86 [-1.16, -0.56]
Station 87	-2.25	1.25	-0.88 [-1.16, -0.60]
Station 86	-2.19	1.31	-0.88 [-1.16, -0.60]
Station 85	-2.26	1.3	-0.86 [-1.16, -0.56]
Station 84	-2.21	1.3	-0.82 [-1.16, -0.49]
Station 83	-2.25	1.29	-0.86 [-1.16, -0.56]
Station 82	-2.28	1.35	-0.86 [-1.16, -0.56]
Station 81	-2.29	1.35	-0.86 [-1.16, -0.56]
Station 80	-2.3	1.43	-0.88 [-1.16, -0.60]
Station 79	-2.27	1.36	-0.86 [-1.16, -0.56]
Station 78	-2.31	1.44	-0.86 [-1.16, -0.56]
Station 77	-2.2	1.31	-0.84 [-1.16, -0.53]
Station 76	-2.22	1.29	-0.86 [-1.16, -0.56]
Station 75	-2.26	1.26	-0.88 [-1.16, -0.60]
Station 74	-2.23	1.39	-0.86 [-1.16, -0.56]
Station 73	-2.13	1.38	-0.88 [-1.16, -0.60]
Station 72	-2.26	1.85	-0.88 [-1.16, -0.60]
RE Model for Subgroup (Q = 0.25, df = 32, p = 1.00; I^2 = 0.0%)			-0.87 [-0.92, -0.82]
**West Yakutat**			
Station 139	-0.91	0.99	-0.80 [-1.16, -0.45]
Station 138	-0.91	1.04	-0.80 [-1.16, -0.45]
Station 137	-0.93	1.07	-0.73 [-1.13, -0.32]
Station 136	-0.99	1.01	-0.69 [-1.12, -0.26]
Station 96	-1.32	1.11	-0.86 [-1.16, -0.56]
Station 95	-0.91	1.01	-0.77 [-1.15, -0.39]
Station 94	-0.93	1.03	-0.75 [-1.14, -0.35]
Station 93	-1.05	1.04	-0.82 [-1.16, -0.49]
Station 92	-1.04	0.96	-0.88 [-1.16, -0.60]
Station 91	-1.54	0.77	-0.88 [-1.16, -0.60]
Station 90	-1.63	0.92	-0.88 [-1.16, -0.60]
Station 89	-3.6	1.86	-0.88 [-1.16, -0.60]
RE Model for Subgroup (Q = 1.57, df = 11, p = 1.00; I^2 = 0.0%)			-0.83 [-0.93, -0.74]
**East Yakutat/Southeast**			
Station 149	0.13	0.32	0.38 [-0.17, 0.93]
Station 148	0.06	0.25	0.09 [-0.50, 0.68]
Station 145	0	0.39	0.07 [-0.52, 0.66]
Station 144	0.1	0.31	0.38 [-0.17, 0.93]
Station 143	0.1	0.25	0.30 [-0.26, 0.86]
Station 142	0.11	0.26	0.30 [-0.26, 0.86]
Station 108	0.07	0.29	0.34 [-0.22, 0.90]
Station 107	0.11	0.28	0.34 [-0.22, 0.90]
Station 106	0.07	0.22	0.34 [-0.22, 0.90]
Station 105	0.1	0.23	0.49 [-0.02, 1.01]
Station 104	0.05	0.32	0.24 [-0.33, 0.82]
Station 103	-0.01	0.33	-0.09 [-0.68, 0.50]
Station 102	0.02	0.3	0.09 [-0.50, 0.68]
Station 101	0.02	0.39	0.07 [-0.52, 0.66]
Station 100	0.07	0.35	0.16 [-0.42, 0.75]
Station 99	0.11	0.25	0.44 [-0.10, 0.97]
Station 98	-0.3	0.73	-0.32 [-0.88, 0.24]
Station 97	-0.6	0.61	-0.73 [-1.13, -0.32]
RE Model for Subgroup (Q = 27.53, df = 17, p = 0.05; I^2 = 40.1%)			0.15 [-0.02, 0.31]
RE Model for All Studies (Q = 232.65, df = 72, p = 0.00; I^2 = 78.6%)			-0.65 [-0.74, -0.57]

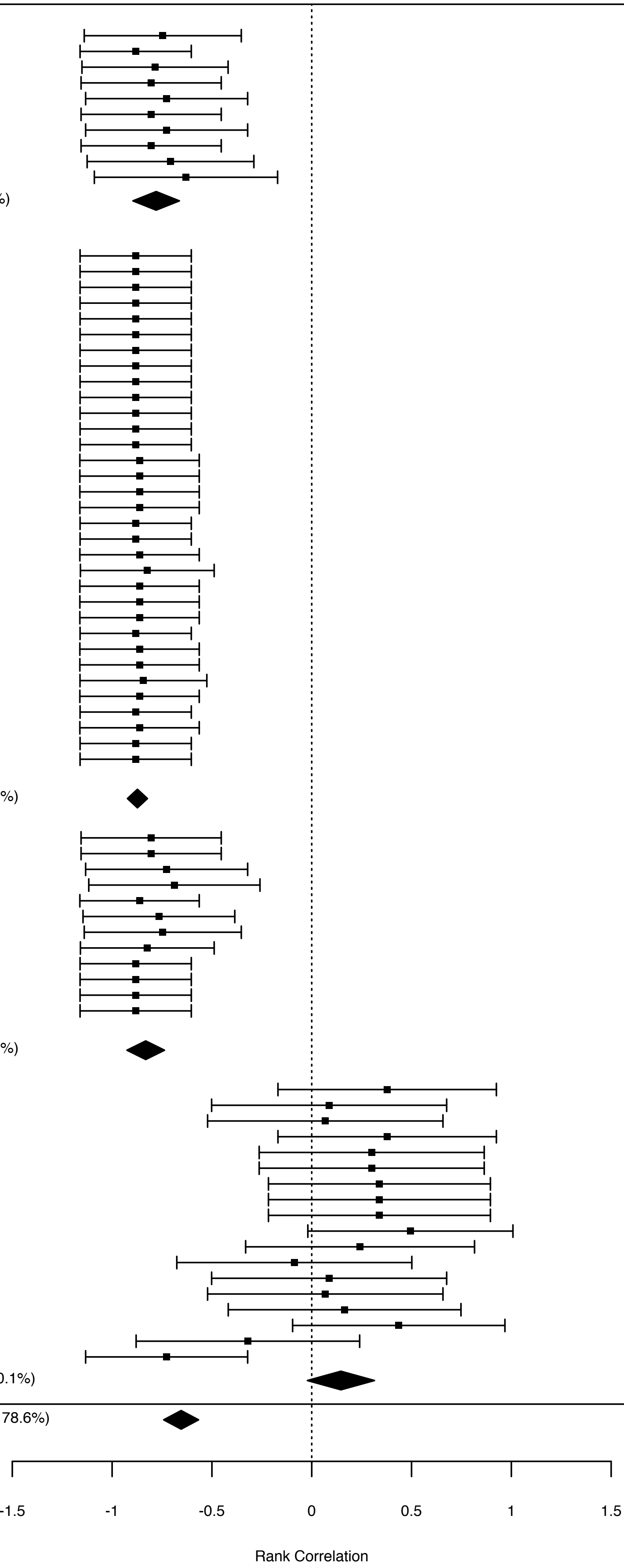

Supplement: Supplementary file 7 [file ECE3-8-12308-s007.pdf]
